# Elevated C-reactive protein and complement C3 levels are associated with preterm birth: a nested case–control study in Chinese women

**DOI:** 10.1186/s12884-020-2802-9

**Published:** 2020-02-27

**Authors:** Shengzhu Huang, Jiarong Tian, Chaoqun Liu, Yu Long, Dehao Cao, Luyun Wei, Xiujuan Zhu, Ruiqiang Tang, Weiwu Liu, Dingyuan Zeng, Mujun Li, Xiaobo Yang, Zengnan Mo

**Affiliations:** 1 Center for Genomic and Personalized Medicine, Guangxi Medical University, Nanning, 530021 Guangxi China; 2 Guangxi key Laboratory for Genomic and Personalized Medicine, Nanning, 530021 Guangxi China; 3 Guangxi Collaborative Innovation Center for Genomic and Personalized Medicine, Nanning, 530021 Guangxi China; 4 School of Public Health, Guangxi Medical University, 22 Shuangyong Road, Nanning, 530021 Guangxi China; 5 Department of Occupational Health and Environmental Health, School of Public Health of Guangxi Medical University, Nanning, 530021 Guangxi China; 6 Department of Gynecology and Obstetrics, First Affiliated Hospital of Guangxi Medical University, Nanning, 530021 Guangxi China; 7 Department of Obstetrics, Maternal & Child Health Hospital of Yulin, Yulin, Guangxi China; 8 Department of Reproductive Center, First Affiliated Hospital of Guangxi Medical University, Nanning, 530021 Guangxi China; 9 Department of Gynecology and Obstetrics, Maternal & Child Health Hospital of Liuzhou, Liuzhou, Guangxi China; 10 Institute of Urology and Nephrology, First Affiliated Hospital of Guangxi Medical University, Nanning, 530021 Guangxi China

**Keywords:** C-reactive protein, Complement C3, Preterm birth, First trimester, Nested case-control study

## Abstract

**Background:**

Currently, there are many studies researched the associations between maternal serum inflammatory indicators (i.e. ferritin, C-reactive protein [CRP], C3 and C4) and preterm birth (PTB). The results, however, are inconsistent. Therefore, the aim of this study was to estimate the relationship between maternal serum inflammatory indicators and PTB in a nested case-control (NCC)study.

**Methods:**

A NCC study was conducted by Guangxi Birth Cohort Study which enrolled a total of 6203 pregnant women between 5^0/7^ and 34^6/7^ weeks of gestational age (wGA) from six cities in China between 2015 and 2016. There were 206women who delivered preterm (< 37^0/7^ wGA), and 412 women who delivered term birth, those women were matched by maternal age, birth place, gender of infants, and wGA at blood collection.

The inflammatory indicators were quantified by immunoturbidimetric methods.

**Results:**

Highest quartile concentrations of all inflammatory indicators were determined versus median. After adjusting for maternal age, high levels of CRP (CRP > 16.60 mg/L) are related to the risk of PTB (OR = 2.16, 95% CI: 1.02–4.56, *p* = 0.044) in the first trimester. The association of C3 was extremely related to those who delivered PTB (OR = 2.53, 95% CI: 1.14–5.64, *p* = 0.023) in the first trimester. Moreover, no significant associations were found in C4 (*p* = 0.079) and ferritin (*p* = 0.067) between PTB.

**Conclusions:**

Elevated concentrations of CRP and C3 in the first trimester were associated with increased risk of PTB. Inflammatory indicators may act a pivotal part in early diagnosis and prognosis of PTB.

## Background

Preterm birth (PTB), an abnormal pregnancy status, defined as delivery before 37 weeks of gestation [1]. It is a major cause of mortality of premature infants and can result in a series of long-term complications in survivors. Complications of PTB are the main reasons which cause deaths of children under 5 years of age, which caused nearly 1 million deaths in 2015. The rate of PTB across 184 countries ranges from 5 to 18% in newborns [2]. In addition, high financial costs are incurred by PTB in terms of immediate neonatal intensive care, and subsequent on-going and long-term complex health care may also lead to heavy financial burdens. However, the causes and mechanisms of PTB remain unknown which limit the prediction and prevention of PTB.

Subclinical infections and chronic inflammation may be the main factors of PTB during pregnancy [3, 4]. In addition, 25–40% of these deliveries were caused by infections [5]. Urinary tract infections, bacterial vaginosis and human immunodeficiency virus have been correlative with increased risk of PTB [6]. However, these findings are often relatively late, and PTB is inevitable in the process. Identification of early markers of PTB may help to successfully intervene. Although C-reactive protein (CRP), C3, C4 and ferritin have been associated with PTB [7–10], many of them only analyze separately rather than combine together. In addition, most studies are limited to a single trimester. Importantly, the development of PTB in southwest Chinese women has not been reported yet. Specifically, more accurate methods to identify women at risk of PTB during pregnancy and sufficiently early in gestation are needed to allow clinical intervention.

To better understand the changes in maternal autoimmunity during pregnancy and find appropriate markers in early pregnancy to prevent the occurrence of preterm birth, we measured the concentrations of CRP, C3, C4 and ferritin before 32 wGA, and estimated the relationship between maternal serum inflammatory indicators and PTB in a nested case-control study in Southwest Chinese women.

## Methods

### Study design and subjects

The NCC study was established in 2015 which based on Guangxi Birth Cohort Study. Briefly, participants were enrolled from eight Maternal & Child Health Hospitals in six cities in Guangxi, China. The participants attended obstetric screening during gestation between July and September 2015. Participants from these Maternal & Child Health Hospitals provided blood samples before 32 weeks or less at a time point to test for serum biomarkers. All participants provided written informed consent and the study was approved by the Medical Ethics and Human Subject Committee of First Affiliated Hospital of Guangxi Medical University (ID: 2015(028)).

The cohort study was conducted among 6203 participants. All participants were followed up until the end of pregnancy. As a result, 5541 participants had pregnancy outcomes and 662 participants lost follow-up. All participants completed a large-scale integrated census through face-to-face interviews and professional maternity checkup by a professional gynecologist. The characteristics of maternal height, pre-pregnancy weight and date of last menstrual period were obtained by self-report. Maternal body mass index (BMI) was calculated. by the pre-pregnancy body weight (kg) divided by the square of height (m^2^).

**Fig. 1:**
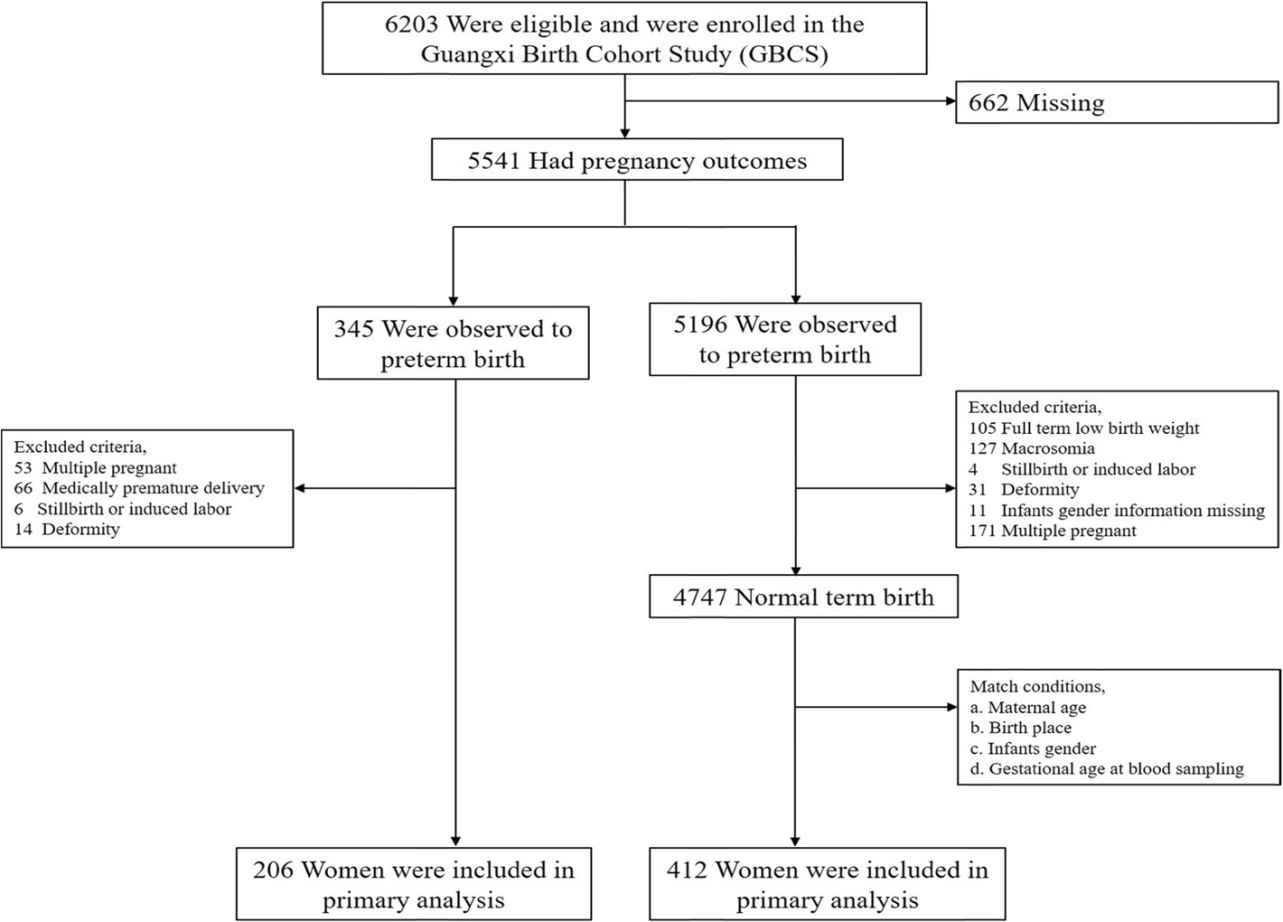
Flow chart for selection of study participants. Flow chart displays a basic process of the nested case-control study conducted by Guangxi Birth Cohort Study. Those exclusion criteria and match conditions are distinguished in different steps

Exclusion criteria included preexisting medical disorders (i.e. preexisting diabetes, gestational diabetes, autoimmune disorders, current cancer diagnosis, human immunodeficiency virus, and hepatitis), pregnant women who had medically premature delivery, stillbirth or induced labor, deformity, macrosomia, full term low birth weight neonatal and multiple pregnancy (Fig. 1).

Eligibility criteria included women who met all of the criteria listed above and delivered live singleton PTB (< 37 wGA) were selected as PTB. Pregnant women who gave singleton neonate at term (≥ 37 weeks and ≤ 42 weeks). The women were 18–45 years.

According to the above criteria, 206 women were selected in the PTB group. These women were matched by maternal age, birth place, gender of infants, and wGA at blood collection in 1:2 ratios and women who delivered term birth infants were selected as control. Finally, 412women were selected as control.

### Biochemical analysis

About 5 ml overnight fasting venous blood was drawn from the pregnant women when they had a pregnancy test in the morning, and the blood was collected before 32 wGA. Then the blood was centrifuged and stored at − 80 °C until it was sent to the First Affiliated Hospital of Guangxi Medical University for analysis 2 years later. It was the same in the preterm and term group. All samples were analyzed by the same method to ensure reliable results.

CRP level (mg/L) was measured on the HITACHI 7600 biochemistry analyzer (Hitachi Crop, Tokyo, Japan) by using the immunoturbidimetric methods with CRP assay kits (Randox Laboratories Ltd., Co Antrim, UK), which intra- and inter-batch CV were 2.37 and 6.02%, respectively. Both C3 and C4 (g/L) levels were measured by immunoturbidimetric technique using the HITACHI 7600 biochemistry analyzer (Hitachi Crop, Tokyo, Japan) with an intra-assay CV of less than 6% and inter-assay relative range of less than 8%, which use the Reagent Kits for C3 and C4 Test (Zhicheng Biological Technology Co. Ltd.; Shanghai, China). Serum ferritin (ng/mL) level was measured with an automatic electrochemical luminescence immunoassay on COBAS 6000 system E601(Elecsys module) analyzer (Roche Diagnostic, Gmbh Mannheim, Germany) with an inter-assay coefficients of variation (CV) of 4.5%. The managing obstetricians were blinded to the results of the serum CRP/C3/C4/ferritin concentrations.

### Data sources

Laboratory records and analysis of maternal serum CRP, C3, C4 and ferritin biomarkers were obtained from the Laboratory of the First Affiliated Hospital of Guangxi Medical University. The subjects’ data on perinatal and birth outcomes were collected from electronic medical records of 8 Maternal & Child Health Hospitals. The perinatal data included maternal features, pregnancy, parturition and neonate characteristics at birth. All birth reports were diagnosed by professional obstetricians and entered into the maternal and child health care system. All pregnant women’s labor data were exported from this system.

### Statistical analyses

All analyses were performed by IBM SPSS 19.0. The continuous variables were performed by Student’s test, and categorical variables were examined by chi-square statistics between two groups.

We evaluated the associations between maternal serum levels of CRP, C3, C4, ferritin and the risk of PTB by using conditional logistic regression. Concentrations of serum inflammatory indicators were examined continuously and as quartiles. Effect estimates were presented as odds ratios (ORs) with 95% confidence intervals (95%CIs). Initial unadjusted analyses were conducted and subsequently adjusted with covariates showing association with both infant PTB and maternal pathology. Potential confounding factor for inclusion in the final model was maternal age. Statistical test were two-tailed, and *p* value < 0.05 was considered statistical significance.

## Results

### Study population

Figure 1 showed the flow chart of the study population. A total of 6203 pregnant women were recruited in GBCS, and 662 women lost follow-up. And 5541 pregnant women were followed-up till delivery. PTB was observed in 345 women, and 4747 had normal term birth (TB). According to the inclusion and exclusion criteria, we selected 206 cases and 412 controls in our research.

The baseline characteristics of the pregnant women at follow-up were shown in Table 1. None of the sociodemographic characteristics differed significantly between women who experienced PTB and TB (*p* > 0.05). In addition, compared with the TB group, the PTB group had significantly higher levels CRP (*p* = 0.029). Moreover, no significant difference in ferritin (*p* = 0.161), C3 (*p* = 0.255) and C4 (*p* = 0.451) were found between the two groups (Table 2).

**Table 1:** General characteristics of the study population

N	Preterm n(%) 206 (100)	Term n(%) 412 (100)	*P* ^a^ value
Maternal age (years) [mean ± SD]	28.35 ± 5.32	28.25 ± 4.70	0.808
Maternal age (years)			0.732
< 25	47 (22.8)	99 (24.0)	
25–34	130 (63.1)	264 (64.1)	
≥ 35	29 (14.1)	49 (11.9)	
Neonatal gender			1.000
Male	121 (58.7)	242 (58.7)	
Female	85 (41.3)	170 (41.3)	
Gestational age at blood sampling (weeks)			1.000
< 14	68 (33.0)	136 (33.0)	
14–27	116 (56.3)	232 (56.3)	
≥ 28	22 (10.7)	44 (10.7)	
pre pregnancy BMI ^b^			0.060
< 18.5	60 (29.6)	98 (24.0)	
18.5–23.9	120 (59.1)	280 (68.4)	
≥ 24.0	23 (11.3)	31 (7.6)	
Literacy			0.647
Primary	7 (3.4)	18 (4.4)	
Secondary	122 (59.5)	255 (61.9)	
Graduate and above	76 (37.1)	139 (33.7)	
Household yearly income (yuan)			0.747
< 50,000	94 (45.6)	197 (47.8)	
≥ 50,000	71 (34.5)	143 (34.7)	
Unclear	41 (19.9)	72 (17.5)	
Parity			0.586
Nullipara	124 (60.8)	241 (58.5)	
Multipara	80 (39.2)	171 (41.5)	
Abortions			0.610
0	101 (49.5)	195 (47.3)	
≥ 1	103 (50.5)	217 (52.7)	
Second-hand smoking			0.930
Yes	25 (12.1)	49 (11.9)	
No	181 (87.9)	363 (88.1)	
Maternal reproductive tract infection			0.935
Yes	29 (14.1)	59 (14.3)	
No	178 (85.9)	353 (85.7)	

^a^ *P* values were determined by using the chi-square test

^b^ BMI body mass index;

**Table 2:** The comparison of serum CRP, C3, C4 and ferritin concentrations between preterm and term

	Preterm(*n* = 206)	Term(*n* = 412)	*P* ^a^ value
Serum CRP (mg/L)	18.38 ± 13.72	16.05 ± 9.24	0.029
Serum C3 (mg/L)	1334.03 ± 218.76	1314.22 ± 216.50	0.287
Serum C4 (mg/L)	271.25 ± 92.15	265.57 ± 86.12	0.451
Serum ferritin (ng/ml)	80.99 ± 85.14	71.37 ± 67.22	0.159

^a^ *P* values were determined by using the Student’s T test;

### Study outcomes

**Fig. 2:**
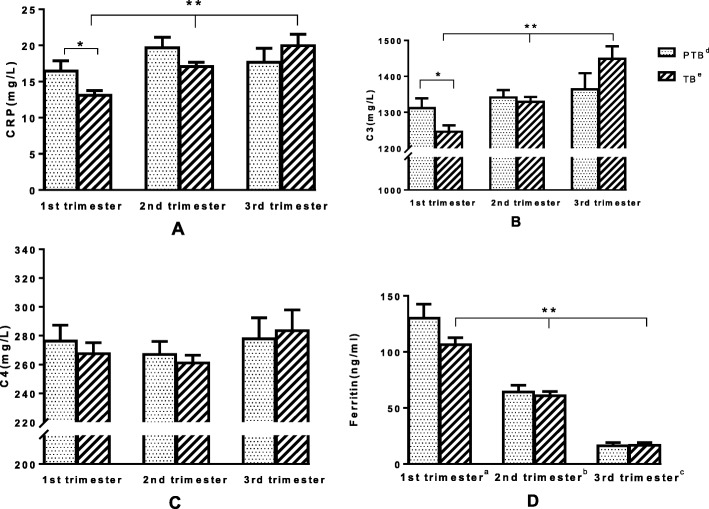
The comparison of Serum CRP, C3, C4 and ferritin among three different trimesters. Distribution of inflammatory factors including C-reactive protein (CRP), C3, C4 and ferritin across different trimesters (*p* < 0.01 for CRP, C3 and ferritin in term birth group at three different trimesters). The first trimester defined as gestational age less than 14 weeks, of which PTB group (*n* = 68), TB group (*n* = 136). In the first trimester, serum concentrations of CRP and C3 in PTB group were significantly higher than the TB group (group effect, *p* < 0.05). For C4 and ferritin, there was no significant difference between preterm and term group (group effect, *p* > 0.05). The second trimester gestational age ranged from 14 to 27 weeks, with the PTB group (*n* = 116) and the TB group (*n* = 232). The third trimester defined as the gestational age of 28 weeks or more, the PTB group (*n* = 22), the TB group (*n* = 44). In any stage of the second and third trimester, the inflammation indicators (i.e. ferritin, CRP, C3 and C4) were not significantly difference with PTB

The result indicated that, with pregnancy progressed, the levels of ferritin dropped while CRP and C3 levels increased among the TB group (Fig. 2). The concentrations of ferritin, CRP and C3 of the term group were significantly different from those of the PTB group during pregnancy (trimester effect, *p* < 0.01). The blood biochemical parameters for two groups in the different trimesters were shown in Fig. 2. In the first trimester, serum CRP and C3 were remarkably higher in the PTB than in TB group (group effect, *p* < 0.05). By contrast, no significant differences were found in serum C4 and ferritin between PTB and term groups (group effect, *p* > 0.05). In PTB group, the inflammation indicators were not significantly different between the second and the third trimester.

The relationship between maternal ferritin, CRP, C3 and C4 levels and the risk of PTB in the first trimester are shown in Table 3. Compared with the median of CRP, in the unadjusted analyses, the OR of PTB in the highest CRP quartile was 2.13 (95% CI: 1.01–4.50, *p* = 0.048). Furthermore, after adjusted age, the OR of PTB in the highest CRP quartile was 2.16 (95% CI: 1.02–4.56, *p* = 0.044). For C3, the unadjusted OR was 2.28 (95% CI: 1.06–4.91, *p* = 0.034), while the adjusted OR was 2.53 (95% CI: 1.14–5.64, *p* = 0.023).

**Table 3:** Association of maternal CRP, C3, C4 and ferritin levels and the risk of PTB in the first trimester

	PTB	Term		Unadjusted		Adjusted	
	(n)	(n)	*P*^a^	OR (95%CI)	*P*^b^	OR (95%CI)	*P*^c^
CRP			0.043				
P50 < 11.40	24	68		1	–	1	–
P50 - P75 (11.40–16.60)	20	34		1.67 (0.80–3.51)	0.175	1.73 (0.81–3.67)	0.154
P75 > 16.60	24	34		2.13 (1.01–4.50)	0.048	2.16 (1.02–4.56)	0.044
C3			0.045				
P50 < 1222.00	26	68		1	–		
P50 - P75 (1222.00–1369.00)	16	34		1.32 (0.60–2.90)	0.493	1.45 (0.64–3.30)	0.370
P75 > 1369.00	26	33		2.28 (1.06–4.91)	0.034	2.53 (1.14–5.64)	0.023
C4			0.178				
P50 < 258.50	30	68		1	–	1	–
P50 - P75 (258.50–309.00)	13	35		0.85 (0.38–1.87)	0.679	0.85 (0.38–1.88)	0.689
P75 > 309.00	25	33		1.77 (0.88–3.57)	0.111	1.89 (0.93–3.85)	0.079
ferritin			0.107				
P50 < 84.07	27	68		1.00	–	1	–
P50 - P75 (84.07–138.85)	17	34		1.27 (0.62–2.63)	0.512	1.29 (0.62–2.67)	0.492
P75 > 138.85	24	34		1.92 (0.92–4.04)	0.084	2.03 (0.95–4.34)	0.067

*P*^a^ Kruskal Wallis test

*P*^b^ unadjusted conditional logistic regression analysis

*P*^c^ adjusted age conditional logistic regression analysis

## Discussion

In our study, the levels of inflammation biomarkers, namely, CRP, C3, C4 and ferritin in the peripheral blood serum were investigated during pregnancy. Elevations in the average levels of CRP and C3 in the first trimester were associated with increasing occurrence of PTB.

CRP, an acute phase reactant in the innate immune response, is a nonspecific biomarker of inflammation that is usually used as a marker for diagnosis of many inflammatory, infective, and malignant conditions [11]. Elevated concentrations of CRP in peripheral circulation were related to the existence of intrauterine infection [12]. Although many studies have shown that increasing levels of CRP are linked to PTB [13], it is the first survey of pregnant women in Southwest China.

In this study, we found that highly expressed CRP and PTB were remarkably association in the first trimester but no relationship was found in the second trimester (Table 4) and the third trimester (Table 5) according to the conditional logistic regression analysis. Our results are consistent with some [14], but not all [15, 16] previous reports. The absence of association between maternal CRP concentrations with subsequent preterm birth also suggested that our positive findings may have an origin other than a generalized inflammatory response. Although no obvious correlation was found between CRP levels between PTB in the second and third trimester, changes in CRP concentrations throughout pregnancy were consistent with the results of Ferguson et al. [15] and de Oliveira et al. [14].

**Table 4:** Association of maternal CRP, C3, C4 and ferritin levels and the risk of PTB in the second trimester

	PTB	Term		Unadjusted		Adjusted	
	(n)	(n)	*P*^a^	OR (95%CI)	*P*^b^	OR (95%CI)	*P*^c^
CRP			0.211				
P50 < 14.9700	48	115		1.00	–	1.00	–
P50 - P75 (14.9700–20.8375)	35	59		1.48 (0.83–2.64)	0.179	1.50 (0.84–2.66)	0.173
P75 > 20.8375	32	57		1.39 (0.77–2.50)	0.275	1.35 (0.75–2.44)	0.321
C3			0.259				
P50 < 1313.00	51	115		1.00	–	1.00	
P50 - P75 (1313.00–1457.00)	30	59		1.18 (0.65–2.12)	0.592	1.15 (0.63–2.08)	0.650
P75 > 1457.00	34	56		1.37 (0.76–2.46)	0.296	1.33 (0.74–2.41)	0.342
C4			0.336				
P50 < 252.50	51	115		1.00	–	1.00	–
P50 - P75 (252.50–305.25)	31	58		1.20 (0.69–2.08)	0.514	1.18 (0.68–2.05)	0.558
P75 > 305.25	33	58		1.33 (0.74–2.39)	0.349	1.25 (0.69–2.29)	0.463
ferritin			0.943				
P50 < 39.3550	59	115		1.00	–	1.00	–
P50 - P75 (39.3550–82.7550)	26	59		0.83 (0.45–1.54)	0.558	0.81 (0.44–1.53)	0.522
P75 > 82.7550	30	57		1.02 (0.56–1.86)	0.949	1.03 (0.56–1.88)	0.922

*P*^a^ Kruskal Wallis test

*P*^b^ unadjusted conditional logistic regression analysis

*P*^c^ adjusted age conditional logistic regression analysis

**Table 5:** Association of maternal CRP, C3, C4 and ferritin levels and the risk of PTB in the third trimester

	PTB	Term		Unadjusted		Adjusted	
	(n)	(n)	*P*^a^	OR (95%CI)	*P*^b^	OR (95%CI)	*P*^c^
CRP			0.964				
P50 < 17.77	12	22		1.00	–	1.00	–
P50 - P75 (17.77–24.08)	4	11		0.68 (0.20–2.34)	0.537	0.77 (0.21–2.85)	0.700
P75 > 24.08	6	10		1.19 (0.35–4.00)	0.779	1.22 (0.36–4.18)	0.748
C3			0.228				
P50 < 1443.00	14	22		1.00	–	1.00	
P50 - P75 (1443.00–1640.00)	6	11		0.69 (0.19–2.57)	0.583	0.64 (0.17–2.38)	0.507
P75 > 1640.00	2	10		0.17 (0.02–1.77)	0.140	0.16 (0.02–1.65)	0.124
C4			0.433				
P50 < 255.00	8	22		1.00	–	1.00	–
P50 - P75 (255.00–349.00)	9	11		3.11 (0.76–12.7)	0.115	2.80 (0.66–11.78)	0.162
P75 > 349.00	5	10		1.68 (0.44–6.41)	0.449	1.61 (0.41–6.26)	0.493
ferritin			0.702				
P50 < 10.34	9	22		1.00	–	1.00	–
P50 - P75 (10.34–18.81)	9	11		1.91 (0.62–5.86)	0.258	1.66 (0.54–5.15)	0.378
P75 > 18.81	4	10		0.94 (0.24–3.78)	0.935	0.58 (0.12–2.82)	0.500

*P*^a^ Kruskal Wallis test

*P*^b^ unadjusted conditional logistic regression analysis

*P*^c^ adjusted age conditional logistic regression analysis

The pro-inflammatory function of CRP includes the induction of cytokines and tissue factor in the monocytes [17]. The monocyte plays a pivotal role in maternal immunological adaptation in which maternal immunity facilitates the maintenance of gestation and protects maternity against infection [18]. This phenomenon may explain the elevated gain of CRP concentrations with wGA in the term group.

The complement, a central part of non-specific and specific immune system, has three renowned physiologic activities, including host defence against infection, bridging interface between non-specific and specific immunity, and phagocytosis cleaning action [19, 20]. About 30 serum complement proteins have been identified and can be activated by classical, alternative and lectin pathways [21]. Of these complement members, C3 and C4 play a pivotal role in activation pathways, which play strongly role as host defence proteins [22]. This phenomenon is particularly obvious in the first trimester, with intense tissue turnover and complement activation which reflects and aggravates the state of inflammation during normal pregnancy [23]. Complement system was activated and involved in inflammation during pregnancy. The relationship between complement activation and PTB has been paid remarkable attention due to the important relationship of the complement system with inflammation [9, 24]. Therefore, our study examined the overall C3 and C4 to verify their association with PTB in the cohort.

In early pregnancy, the elevated serum levels of C3 in pregnant women were positively correlated with PTB. This observation is interesting because infection and inflammation are strongly causal risk factors of PTB [25]. We examined the quartile levels of C3 in women with PTB according to the categories of wGA. The results showed a strong relationship between the highest quartile concentrations of C3 and women who had PTB below 14 wGA. The relationship of C3 concentrations with the development of PTB during early pregnancy was significantly relevant, which was consistent with published findings [22, 26]. Serum levels of C3 and C4 as well as total complement activity in normal pregnancy were gradually increased [27] and these parameters were even further elevated in participants with preterm birth which may due to the activation of the inflammatory response in the maternal peripheral circulation. However, this upward trend appeared only in the first and second trimesters. The decrease in complement levels in the third trimester may indicate that increased utilization because of antigen-antibody reactions, whereas decreased complement activity would be compatible with either decreased production or increased utilization. As for C3, which decreased during pregnancy, and the most probable mechanism underlying this phenomenon may be due to the decreased production of C3 by the liver. No evidence that demonstrate why these diverse changes are observed in women who had PTB has been reported.

Although the complement system was activated and involved in inflammation during pregnancy, no significant association was found between C4 and PTB. In addition, the trends of C4 concentrations varied in our study varied, probably because our study was focused on asymptomatic pregnant women, whereas previous studies focused on pregnant women with preeclampsia or systemic lupus erythematosus [28]. In addition, other studies on C4 were performed by using neonatal umbilical cord blood and/or newborn’s blood [29] while we performed on the blood of pregnant women.

Ferritin is an acute phase reactant, and elevated level of this molecule has been associated with acute/chronic infection, inflammation, neurodevelopmental disability and malignant diseases [30]. Previous reports [7] have shown that changes in maternal ferritin levels were associated with PTB, and increased odds of PTB were associated with ferritin levels >75th percentile. Although no significant association was found between serum ferritin and PTB, trends in ferritin throughout pregnancy were consistent with other studies. The conflicts may be attributed to the diversities in the populations studied, study design, timing of blood collection and incomplete or no control for confounding.

To sum up the appeal, in Fig. 2, we found that the concentrations of CRP, C3, C4 and ferritin were higher in the early and middle trimester than control group and the phenomenon was reversed in the third trimester. As we know, pregnancy is an autoimmune process, the presence of immune defense response occurs maternal self-protection mechanism, and in the third trimester of pregnancy, the immune defenses decline in the case group compared with the control group, thus preterm birth.

In a systematic review, similar results were found in populations in the United States, Iran, and Denmark [31]. Specifically, our study found that elevated CRP and C3 levels in early pregnancy increased the risk of PTB, while ferritin and C4 levels were not associated with PTB throughout pregnancy. One report found no association between CRP and PTB in Caucasian and African-American populations [15]. This may be the result of a combination of race, genes, dietary habits, environmental factors and inflammatory factors, which needs further research to confirm.

We chose the primary outcome of pregnancy-associated PTB with evidence of maternal factors, rather than the diagnosis of PTB, to assess whether therapy would prevent serious complications rather than merely modify diagnostic findings.

Our study had several strengths. The NCC study design provided an opportunity to include all PTB infants in the study. Importantly, the NCC study design can be clearly illustrated the causal link between serum inflammatory factors and prematurity during the first trimester of pregnancy. To our knowledge, following a MEDLINE search, the current study is the first study to investigate whether inflammatory indicators are associated with Southwest Chinese normal pregnant women. In addition, all participants also adjusted for other potential risk factors, such as maternal age and pre-pregnancy BMI. It is known that the concentration of CRP in the peripheral circulation is associated with high BMI and other obesity markers [32, 33]. Thus, pre-pregnancy BMI was not considered in the logistic regression model. Maternal reproductive tract infections are closely related to inflammation. We detect indicators are inflammatory markers and therefore do not correct it.

The current study also has some limitations. Firstly, maternal serum samples were collected at one time only and might not accurately reflect the change of CRP and C3 during the whole pregnancy in our study. Secondly, the study was limited by the wide range of wGA of blood drawn (between 6 and 32 wGA). A narrower range of wGA within the data points will help to assess the association of complement markers with more predictive biomarkers.

Further predictive analysis requires a large-scale, multicenter, prospective cohort study to investigate the predictive value of these inflammatory markers in maternal blood for preterm delivery in asymptomatic pregnant women and to develop a valuable multivariate predictive model.

## Conclusion

In summary, a NCC study of 206 cases and 412 controls subjects was performed higher levels of CRP and C3 early in pregnancy were associated with an increased risk of PTB in the first trimester. Our results indicated that a close relationship between early pregnancy inflammatory indicators and PTB in Southwest Chinese women. Further studies are needed to characterize the relationship between inflammatory indicators and long-term outcome using well-defined diagnostic PTB. Additional evaluations are warranted to increase the sample size to target the relationship between high levels of inflammatory indicators and long-term outcome.

How to manage these patients to prevent PTB, however, presents several great challenges. Our research merely found that elevated serum CRP and C3 levels increase the risk of preterm birth, and is not a diagnosis in itself. There are many pathways leading to preterm birth and the prevention of each requires different types of scientific inquiry and clinical strategies, which together encompass a wide array of measurement systems and clinical interventions across many health-care disciplines [34].We need to conduct further studies to confirm whether relevant biomarkers can be used as indicators to diagnose preterm birth.

Quantification of the reference range is difficult to predict which will depend on other clinical, cultural, social and economic factors that operate in each environment. Further success is expected in the coming years as other research findings translate into clinical practice, including new ways to treat intrauterine infections, improved maternal nutrition and lifestyle improvements to reduce maternal stress.

## Data Availability

The datasets used and/or analyzed during the current study are available from the corresponding author on reasonable request.

## Abbreviations

95%CIs: 95% confidence intervals
CRP: C-reactive protein
NCC: Nested case-control
ORs: Odds ratios
PTB: Preterm birth
TB: Term birth
wGA: Weeks of gestational age
